# The Effect of Different Starch Liberation and Saccharification Methods on the Microbial Contaminations of Distillery Mashes, Fermentation Efficiency, and Spirits Quality

**DOI:** 10.3390/molecules22101647

**Published:** 2017-09-30

**Authors:** Katarzyna Pielech-Przybylska, Maria Balcerek, Agnieszka Nowak, Maciej Wojtczak, Agata Czyżowska, Urszula Dziekońska-Kubczak, Piotr Patelski

**Affiliations:** 1 Department of Spirit and Yeast Technology, Institute of Fermentation Technology and Microbiology, Faculty of Biotechnology and Food Sciences, Lodz University of Technology, Wolczanska 171/173, 90-924 Lodz, Poland; maria.balcerek@p.lodz.pl (M.B.); urszula.dziekonska-kubczak@p.lodz.pl (U.D.-K.); piotr.patelski@p.lodz.pl (P.P.); 2 Department of Technical Microbiology, Institute of Fermentation Technology and Microbiology, Faculty of Biotechnology and Food Sciences, Lodz University of Technology, Wolczanska 171/173, 90-924 Lodz, Poland; agnieszka.nowak@p.lodz.pl (A.N.); agata.czyzowska@p.lodz.pl (A.C.); 3 Institute of Food Technology and Analysis, Faculty of Biotechnology and Food Sciences, Lodz University of Technology, Wolczanska 171/173, 90-924 Lodz, Poland; maciej.wojtczak@p.lodz.pl

**Keywords:** starch liberation, ethanol fermentation, malt, hop α-acids, agricultural distillate

## Abstract

The aim of this study was to evaluate the influence of different starch liberation and saccharification methods on microbiological contamination of distillery mashes. Moreover, the effect of hop α-acid preparation for protection against microbial infections was assessed. The quality of agricultural distillates was also evaluated. When applying the pressureless liberation of starch (PLS) and malt as a source of amylolytic enzymes, the lactic acid bacteria count in the mashes increased several times during fermentation. The mashes obtained using the pressure-thermal method and malt enzymes revealed a similar pattern. Samples prepared using cereal malt exhibited higher concentrations of lactic and acetic acids, as compared to mashes prepared using enzymes of microbial origin. The use of hop α-acids led to the reduction of bacterial contamination in all tested mashes. As a result, fermentation of both mashes prepared with microbial origin enzyme preparations and with barley malt resulted in satisfactory efficiency and distillates with low concentrations of aldehydes.

## 1. Introduction

Among the priority activities of the world economy, the major attention is paid to sustainable development, which takes into account the interactions between nature, economy, and society. The distilling industry is very conscious of environmental and social issues and has made significant contributions to energy and waste management improvement. One example that meets the requirements of sustainable development is promoting organic (bio) production, understood as a system of farm management and food production that combines the best environmental practices, protection of natural resources, application of high animal welfare standards, and the manufacturing methods complying with the requirements of consumers who prefer products made using natural substances and natural processes [1].

Nowadays, the production of ethanol from starchy raw materials in agricultural distilleries is based on the use of amylolytic enzymes of microbial origin, i.e., α-amylase (EC 3.2.1.1) and amyloglucosidase (EC 3.2.1.3). However, the production of spirit beverages, such as Scotch, Irish, and American whisky, employs malt enzymes, including α-amylase, β-amylase (EC 3.2.1.2), limit dextrinase (dextrin α-1,6-glucanohydrolase, EC 3.2.1.41), proteases, glucanases, phosphatases, and lipases. Another example is the okovita, produced from grain distillate, which in accordance with the EU Regulation (European Commission, 2008) is defined as a spirit beverage produced exclusively by the distillation of a fermented mash of whole grain cereals and having the organoleptic characteristics of the raw materials used [2].

In the production of most grain spirits, malt enzymes have been replaced with commercial preparations including enzymes of microbial origin, which offer many advantages, such as high levels of activity in a wide temperature and pH range as well as long storage [3,4]. In turn, to maximize the activity of the native enzymes of plant origin (malt enzymes), it is necessary to strictly comply with appropriate temperature and pH conditions during starch hydrolysis, which are lower than those suitable for enzymes of microbial origin [5], that is, 70–75 °C (α-amylase) and 55–65 °C (β-amylase). To preserve the activity of both amylolytic malt enzymes in the mashing process, lower temperatures (not exceeding 50–56 °C) are often used.

The advantage of the malt-based mashing process is the presence of numerous enzymes that hydrolyze cereal starch and non-starch components, thus providing nutrients to the yeast, as well as the occurrence of a wide range of aroma compounds [6]. One of the serious threats to the fermentation process is a low starch hydrolysis temperature, which may promote microbial contamination. In ethanol production, the most common microbial contaminants are lactic acid bacteria (LAB) [7], especially *Lactobacillus*, *Lactococcus*, *Leuconostoc*, and *Pediococcus* strains [8]. LAB ferment carbohydrates, producing lactic and acetic acids. They also compete for nutrients [9] and are resistant to ethanol concentrations above 10% *v*/*v*, low pH (below 3.5), and high temperature, and thus hinder ethanol yield. The study of Muthaiyan et al. [10] shows that the gradual formation of acids during fermentation reduces the lifespan of yeast cells by up to 60%. It has also been shown that the synergistic action of acetic and lactic acids decreases the yeast growth rate, the glucose consumption rate, and ethanol yield [10]. Broda and Leja [11] estimated the microbiological contamination of different unmalted cereal grains (corn, rye, and triticale) to be 4–8 log cfu of LAB per g and approx. 4.5–7.5 log cfu of total viable bacteria per g. On the other hand, O’Sullivan et al. [12] investigated the malt microflora with a focus on LAB, determining the degree of microbial contamination of barley before and during the malting process (wetting and germination), as well as at the end of the process (after drying).

In agricultural distilleries, both pressure-thermal and pressureless (PLS) methods of starch liberation are used in ethanol production. In the pressure-thermal method, raw materials are sterilized by high temperature (150 °C) and pressure (0.5 MPa). In turn, the PLS method applies a temperature of 90 °C (approx. 60 min), 120 °C (approx. 20 min), or 140 °C (approx. 3–4 min). Sterilization is achieved above 120 °C, while a temperature of 90 °C inactivates most viable vegetative forms of microorganisms, except for spores, which are heat-resistant. Nevertheless, secondary microbial contamination is possible in subsequent stages of processing due to the presence of microorganisms in the water, air, and yeast. Distillery equipment can also be a source of microbes [13].

One of the simplest and easiest methods to reduce growth of undesirable microflora, such as lactic bacteria, is lowering the pH of the fermented medium even to less than 4 value (e.g., with sulfuric acid). The optimal pH range for distillery yeast growth varied from 4 to 6. Narendranath and Power [14] found that lowering the pH of the mash to 4 reduces lactic acid production, without yeast growth inhibition. However, lactic acid bacteria, especially Lactobacilli, are usually more resistant to lower than optimal pH and able to grow in the medium with pH even close to 4.0.

The maintenance of microbiological purity could also be aided by the use of natural compounds of plant origin, limiting the development of undesirable microflora, particularly bacteria. Hop compounds exhibit antimicrobial activity, especially effective against bacterial contamination. Hop α-acids inhibit the growth of Gram-positive bacteria, LAB included, by changing the permeability of bacteria cell membranes [15]. The undissociated forms of hop α-acids diffuse through the membrane into the cell and decrease the intracellular pH of the bacteria, which in turn disrupts their nutrient uptake ability and stimulates the extracellular release of toxic metabolites [16]. Furthermore, Rückle and Senn [17] confirmed that hop α-acids do not inhibit the activity of yeast during fermentation process. The combined effect of low pH of mashes and α-hop acids seems to be a good idea to keep mashes safe from bacterial contamination during ethanol fermentation.

Taking into consideration the above assumptions, the aim of our study was to compare and assess the fermentation efficiency and microbial contamination of distillery mashes prepared with amylolytic enzymes of plant origin with those prepared with enzymes of microbial origin, via different methods of starch liberation and saccharification, as well as to evaluate the obtained agricultural distillates.

## 2. Results and Discussion

### 2.1. Chemical Composition of Raw Materials

Unmalted barley grain of the Karakan variety was used as raw material. As a source of amylolytic enzymes for starch hydrolysis, barley Munich malt type 2 was used. The starchy raw materials were analyzed for the content of moisture, reducing and total sugars, starch, and protein, with the results presented in Table 1.

Malted grain was found to have a lower moisture content (*p* < 0.05) than unmalted barley. The final stage of malt production involves drying. Wet malt is kilned to a final moisture content below 10% [18,19]. The malt used in the study showed a low moisture content (43.40 ± 1.42 g/kg), typical for Munich malt [20]. The moisture of unmalted barley of the Karakan variety was consistent with data published in the literature [21]. The content of sugars (reducing, total, and starch) was also measured. The malted grain contained a significantly higher amount of reducing sugars in comparison with the unmalted grain (*p* < 0.05). The percentage of reducing sugars in total sugars in barley grain of the Karakan variety and in barley malt were 10% and 25%, respectively.

### 2.2. Chemical and Microbiological Characteristic of Mashes

The distillery mashes were processed using two methods of starch liberation: pressure-thermal and pressureless. In both methods, the mashing process was carried out using both enzymatic preparations of microbial and plant origin. In order to examine the influence of starch liberation and saccharification methods on the microbial purity of sweet mashes and to assess the effect of hop α-acids on microbial contamination, mashes were prepared in two variants: with and without hop α-acid IsoStab^®^ preparation.

Both sweet and fermented mashes were analyzed for pH and the content of extract, sugars (glucose, maltose, maltotriose, dextrins, and total sugars), and acids (lactic and acetic). In addition, ethanol concentration was determined in fermented mashes. The results are given in Table 2 and Table 3.

In sweet mashes (fermentation time 0 h), the concentration of glucose was higher (*p* < 0.05) in samples prepared by the pressure-thermal method as compared to the PLS method, using enzymes of both microbial and malt origin. Steaming led to profound changes in starch, involving gelatinization and then solubilization, making the subsequent hydrolysis of starch much more efficient [22,23]. Glucose concentration in sweet mashes after steaming was approx. 2.5-fold and 4.5-fold higher for enzymes of microbial origin and malt enzymes, respectively (*p* < 0.05), in comparison with mashes prepared by the PLS method (Table 2). Glucose concentration in sweet mashes was also affected by the enzymes used. Mashing grain with enzymes of microbial origin resulted in a more than 3-fold increase in glucose concentration in the sweet mash (*p* < 0.05) as compared to malt enzymes. This difference is attributable to the higher activity of enzymes of microbial origin. Moreover, malt β-amylase hydrolyzes starch, dextrin, and oligosaccharides to maltose, while amyloglucosidase of microbial origin hydrolyzes the above-mentioned products of α-amylase activity into glucose molecules [4]. Therefore, the concentration of maltose in sweet mashes was 2-fold and 4.5-fold higher (*p* < 0.05) in samples using malt enzymes prepared by the PLS and pressure-thermal methods, respectively. High concentrations of dextrins (*p* < 0.05) were also observed in sweet mashes prepared with malt enzymes, which indicate the low initial saccharification. These mashes were also characterized with high concentrations of dextrins (*p* < 0.05) as a consequence of carrying out the process without the separate saccharification step.

Despite statistically significant differences in concentrations of sugars determined upon completion of fermentation, it can be observed that both mashes prepared with commercial enzyme preparations and with barley malt as a source of amylases have been fermented properly. The lowest amounts of unutilized glucose, maltose, maltotriose, and dextrins were determined in mashes supplemented with hop α-acid preparation (Table 3).

Sweet mashes and mashes during fermentation were examined microbiologically. The results obtained for samples collected once every 24 h are shown in Table 4.

Moreover, before and after fermentation, lactic and acetic acid content was determined in the mashes (Table 2 and Table 3). The main sources of the microbial contaminations of distillery mashes are raw materials, water, and air. Additionally, the yeast and distillery equipment are an important factors in maintaining the proper purity of the process. Good practice is to use yeast disinfection (by sulfuric acid solution, pH 2–2.5) to eliminate undesirable bacterial cells, before mashes inoculation with yeast [24].

It was found that the use of malt in the mashing process greatly increased (*p* < 0.05) LAB and TMB counts. The LAB count was twice as high (*p* < 0.05) as that in the sample treated with enzyme preparations. O’Sullivan et al. [12] investigated that LAB count in kilned barley malt was 3.5 × 10^7^ cfu/g, while unmalted barley contained only 40–50 cfu/g of LAB.

The LAB and TMB counts in the mashes prepared using the PLS method with malt increased several times (*p* < 0.05) after the first day of fermentation, from 2.00 ± 0.10 to 8.12 ± 0.46 log cfu/mL and from 2.72 ± 0.15 to 7.47 ± 0.32 log cfu/mL (*p* < 0.05), respectively, and afterwards remained at the same level until the end of fermentation (*p* > 0.05). The mashes obtained using the pressure-thermal method and malt enzymes exhibited a similar pattern, with the LAB and TMB counts doubling (*p* < 0.05) after 24 h, and reaching 7.18 ± 0.71 and 7.87 ± 0.69 log cfu/mL at the end of the process, respectively. Broda and Leja [11] determined the LAB and TMB counts in distillery mashes. Before fermentation microbial contamination of different sweet mashes was 2–6 log cfu/mL (LAB) and 3–6 log cfu/mL (TMB), and after 72 h of fermentation increased to 5–8 log cfu/mL and 6.5–8 log cfu/mL for LAB and TMB, respectively. The growth of LAB and the fermentation of sugars result in the secretion of lactic and acetic acids into the medium, among others. HPLC analysis of fermented mashes showed a significantly higher (*p* < 0.05) concentration of lactic acid in samples prepared with malt (7.10 ± 0.21 g/L for the PLS method and 9.58 ± 0.25 g/L for the pressure-thermal method) in comparison with mashes prepared by starch hydrolysis with enzymes of microbial origin (Table 3). This was also true for acetic acid concentration, which was almost 3-fold and 9-fold higher in malt-based fermented mashes (0.62 ± 0.25 g/L for the PLS method and 1.72 ± 0.05 g/L for the pressure-thermal method, respectively) (*p* < 0.05).

The synergistic activity of acetic and lactic acids has been shown to decrease the yeast growth rate, the glucose consumption rate, and ethanol yield [25,26]. Indeed, significantly (*p* < 0.05) lower ethanol content and fermentation efficiency were observed in trials exhibiting more severe bacterial contamination (Table 2, Table 3, Table 4 and Table 5).

In comparison with trials involving enzymes of microbial origin, fermentation efficiency was lower for the PLS and pressure-thermal methods respectively (*p* < 0.05). Sugar intake was higher for all fermentation trials and reached 91.58 ± 3.29% to 95.53 ± 3.21% (*p* < 0.05) due to sugar utilization by both yeast and bacteria (Table 5). Moreover, fermentation of mashes obtained by the PLS method with malt was accompanied by decreased yeast counts. Yeast count declined from 8.41 ± 0.40 log (cfu/mL) at 24 h to 4.18 ± 0.35 log (cfu/mL) at 48 h of fermentation with further reduction to 2.54 ± 0.25 log (cfu/mL) after 72 h (*p* < 0.05). As regards mashes prepared by pressure-thermal treatment, a significant (*p* < 0.05) decrease in the yeast count was only observed between the second and third days of fermentation (from 7.46 ± 0.52 to 6.15 ± 0.47 log cfu/mL).

The most common antibacterial method used in distilleries is acid treatment with sulfuric acid. The sulfuric acid is used to reduce pH of sweet mashes as well as to yeast washing before mashes inoculation. During ethanol fermentation, the pH of mashes drops as a consequence of the removal of buffering compounds and release of organic acids. The major microbial contaminations of distillery mashes are bacteria, mainly lactic acid bacteria. The lactic acid bacteria grow in a wide pH range. Their optimum pH for growth depended on the strain and ranged from 5.5 to 6.9. However, lactic acid bacteria can also grow in the medium with a pH lower than optimum. This is due to a very well-functioning mechanism of pH gradient regulating [27,28].

The results of our study confirmed that lactic acid bacteria growth was observed in mashes with the initial pH of 4.8, which dropped to 3.3–3.7 during ethanol fermentation (Table 3). Therefore, it seems necessary to use additional methods limiting the development of bacteria in the distillery mashes. The plant-based compounds are alternative antibacterial agents for antibiotics. This group is represented, for example, by hop acids, essential oils, lemon extract, phenolic compounds of green tea, etc. [10]. Hop compounds such as hop acids are recognized as safe for human [29].

The use of hop α-acids was very effective in reducing bacterial contamination in mashes obtained by both the PLS and pressure-thermal methods with malt. In the case of PLS, the bacteria count in the mash fermented with hop α-acids was reduced more than 2-fold (from 7.08 ± 0.63 to 3.34 ± 0.33 log cfu/mL, *p* < 0.05) as compared to controls (without hop α-acids), while in mashes obtained with the second method the presence of hop α-acids resulted in a decrease in the LAB count to less than 1 log cfu/mL in comparison with the control sample (7.18 ± 0.71 log cfu/mL) (*p* < 0.05). Reduced bacterial contamination was also observed in mashes prepared using the commercial enzyme preparations. After 72 h, the bacteria count dropped to below 1 log cfu/mL, while bacterial contamination of the control (mash without hop α-acids) reached 3.04 ± 0.30 log cfu/mL (*p* < 0.05) (Table 4).

Rückle and Senn [17] examined the inhibitory potential of hop α-acids to control *Lactobacillus brevis* and *Lactobacillus fermentum* strains during ethanol fermentation of wheat mashes inoculated with 10^7^ cfu/mL of the above-mentioned bacteria strains. The authors observed a reduction in bacteria counts to less than 10^4^ cfu/mL, as well as a more than 90% decline in the concentration of lactic and acetic acids in mashes containing hop α-acids (IsoStab^TM^, Nürnberg, Germany) as compared to controls (without hop α-acids). They noticed that a reduction in bacteria counts to less than 10^6^ cfu/mL efficiently inhibited bacteria metabolism with increasing ethanol yield.

Considering the microbial contamination of starchy raw materials, which are the main cause of distillery mashes infections, the pressure-thermal method, with the use of microbiological origin enzyme preparations, leads to the most favorable results in their elimination. Nevertheless, there is currently a trend to use energy-saving methods and produce bio-organic products. Therefore, changes in technology as well as in materials used in distilleries are being made. The implementation of environmental solutions in distillery technology greatly facilitates the processing of starch raw materials, but the shortcoming is the presence of bacteria that can effectively distort fermentation and reduce the quality of the obtained spirits. The combined use of PLS technology, the enzymes of plant origin, and α-hop acids are in line with the current trend of using natural materials in spirit beverage production.

#### The Discriminant Function Analysis of Microbial Analysis Results

For evaluation of the results of microbial analysis (Table 4), the discriminatory power of the model was calculated, based on the discriminant function analysis. The assessment of discriminating power was estimated on the basis of Wilks’ Lambda coefficient. In the discriminative function, the method of starch liberation, the source of amylolytic enzymes and the time of fermentation, as well as interactions between them were used as the variables. The validity of the model indicated groups that can be differentiated on the basis of the above three dependent variables (Table 6).

The smaller values of the partial Wilks’ Lambda coefficient indicated a stronger contribution of a given variable(s); thus, the variable or the interaction between the variables showed a stronger variation of yeast, lactic acid bacteria, and total bacteria counts. The results of cross-comparison of the source of amylolytic enzymes and the time of fermentation indicated the strongest differentiation of the total mesophilic bacteria, yeast, and lactic acid bacteria counts for which the partial Wilks’ Lambda assumes the smallest value of 0.238, 0.365, and 0.505, respectively. The weakest differentiation occurs only when we compare (without interaction) the method of starch liberation and the time of fermentation, where for yeast count (Wilks’ part. = 0.990) and lactic acid bacteria count (Wilks’ part. = 0.988) no significant differences were observed, respectively. Interactions between method of starch liberation, source of amylolytic enzymes and time of fermentation, resulted in the greatest differences in all counted microorganisms, i.e., TMB (Wilks’ part. = 0.004), LAB (Wilks’ part. = 0.004), and Y (Wilks’ part. = 0.074).

### 2.3. Chemical Composition of the Obtained Distillates

Following ethanol fermentation, distillery mashes contain, along with ethanol, a wide variety of volatile compounds synthesized by the yeast. The presence of other microorganisms (bacteria, wild yeast, or molds) can significantly affect the profile of volatile compounds. Cereals (both unmalted and malted) can also be a source of volatile compounds [6,30]. Analysis of the volatile profile of barley malt has revealed the presence of alcohols, aldehydes, ketones, esters, sulfur compounds, and others [6]. Some of these compounds may be considered as active sensory descriptors of the raw material in the resulting spirit (vodka), while others may be the intermediate products of the reactions occurring during yeast ethanol fermentation, imparting new sensory properties (sensory descriptors of fermentation). During the distillation process, most of them migrate to the produced spirits along with ethanol [31].

Upon completion of the fermentation and ethanol distillation, the obtained distillates were analyzed by gas chromatography, with the results given in Table 7. The evaluation of chemical composition involved carbonyl compounds, acetals, esters, alcohols, and acetic acid.

The obtained results were evaluated using variance analysis (ANOVA, *p* < 0.05) followed by Tukey’s multiple comparison test (Table 7). Significant differences in the concentration of volatile compounds were found between the various methods of starch liberation and hydrolysis (saccharification) (*p* < 0.05). Higher microbial contamination and metabolite concentration adversely affect yeast viability and fermentation activity. High levels of yeast viability and activity enable the reduction of aldehydes to corresponding alcohols, and in particular acetaldehyde to ethanol, while stress factors can significantly inhibit the activity of alcohol dehydrogenase [32]. Aldehyde presence may also be attributed to the oxidation reaction of alcohols [33]. In the obtained distillates, acetaldehyde levels were higher in the control samples of mashes (without hop α-acids) prepared using both the PLS and the pressure-thermal methods (*p* < 0.05). The addition of anti-bacterial hop α-acids to fermentation samples (PLS method) decreased acetaldehyde concentration in the distillates by 63% in the case of mash produced with malt and by 18% in the case of mash produced with commercial enzyme preparations (*p* < 0.05). A similar pattern was observed for the pressure-thermal method, in which acetaldehyde reduction amounted to over 40% (*p* < 0.05). The concentrations of other carbonyl compounds, such as isobutyraldehyde, isovaleraldehyde, 2-methylbutyraldehyde, phenylacetaldehyde, furfural, and 2,3-butanedione were determined and found to decrease in the samples with microbial protection (*p* < 0.05). The presence of isovaleraldehyde and phenylacetaldehyde was only found in the distillates obtained from mashes prepared with malt. Indeed, these two aldehydes are compounds of malt origin. They are formed during malt production as a result of Maillard’s reaction [34]. De Clippeleer et al. [35] reported them in malt and beer samples.

Some bacteria, among others of genera *Lactobacillus* and *Leuconostoc*, are able to form volatile compounds such esters, alcohols, and carbonyl compounds [36]. In the distillates obtained from mashes prepared with using malt as a source of enzymes, ethyl lactate (i.e., ethyl 2-hydroxypropanoate) was presented in higher concentration, but only in trials without microbial protection (*p* < 0.05). Respectively, the presence of a large number of lactic acid bacteria was found in these samples of mashes (Table 4). High level of ethyl lactate, with increasing levels of microbial contamination was also observed in other studies [37].

Other common compounds in alcoholic beverages include acetals, which are rapidly formed in distillates. The most prominent of the latter group is acetaldehyde diethyl acetal (1,1-diethoxyethane), of which the highest levels among whiskies were found in malt whisky. In addition to acetaldehyde diethyl acetal, a number of acetals of higher aldehydes have been determined in spirit beverages [33]. Among determined acetals, in highest concentrations in all tested distillates occurred acetaldehyde diethyl acetal. Moreover, it was observed that distillates obtained from mashes supplemented with hop α-acid preparation contained statistically significantly lower amount of this compound than spirits from analogous mashes without microbial protection (*p* < 0.05). No clear effect of the method of sweet mashes preparation on this acetal concentration was observed. Some of the tested distillates also exhibited small amounts of other acetals, such as isobutyraldehyde diethyl acetal and isovaleraldehyde diethyl acetal (no correlation between the method of mash preparation and origin of applied amylolytic enzymes was observed, *p* > 0.05).

Esters are the most odor-active compounds found in fermented media, the most common are those derived from ethyl alcohol and higher alcohols. Their presence is mainly linked to yeast metabolism during ethanol fermentation, so their concentration in distillates was not significantly affected by the microbiological contamination of mashes. However, in the distillates obtained from mashes prepared with malt, the levels of ethyl acetate, ethyl decanoate, and 2-phenylethyl isobutyrate were significantly higher (*p* < 0.05) as compared to samples obtained from mashes prepared with commercial enzymes of microbial origin.

The main group of volatile compounds consists of higher alcohols, which may be produced via amino acid catabolism or carbohydrate metabolism [32]. This group is represented by 2-methylbutanol, 3-methylbutanol, 2-methylpropanol, 1-propanol, 1-butanol, and 2-phenylethanol. Higher alcohols play an important role in the formation of flavor qualities in spirits, including whisky and others. Malt Scotch whiskies are rich in higher alcohols, whose content often exceeds 2 g/L [38]. According to the recommendations of the Polish Standard [39], the maximum concentration of those compounds in agricultural distillates used for Starka production is 5 g/L absolute alcohol. The highest concentrations of 2- and 3-methylbutanol, 2-methylpropanol, and 2-phenylethanol were reported in distillates obtained from mashes treated with enzymes of microbial origin (*p* < 0.05). Moreover, all tested distillates contained higher alcohols in relatively high concentrations in comparison with spirits obtained in our previous work [40], distilled in an alembic with a column (on a semi-technical scale) and in an industrial 2-column continuous apparatus. Taking into account, that in this work ethanol was distilled from the mashes using a laboratory distillation unit and then distillates (containing from 20 to 23% of ethanol by volume) were refined up to approx. 43 ± 1% in a distillation apparatus equipped with a bi-rectifier unit (dephlegmator according to Golodetz), this may explain the significant differences in concentrations of higher alcohols in the obtained distillates comparing to the ones tested previously [40]. These results indicate that, although the content of higher alcohols is strongly associated with the kind of raw material and yeast used for fermentation [41], the type of apparatus used for distillation and the process parameters can modify their content.

One of the undesirable compounds in spirit distillates is methanol, which is generated through hydrolysis of methylated pectins present in plants and fruits. While methanol does not directly affect the flavor of the distillate, it is subjected to restrictive controls owing to its high toxicity [42]. EU Regulation no. 110/2008 [2] defines acceptable concentrations of methanol in ethyl alcohol of agricultural origin (i.e., rectified spirit), wine spirits, and fruit spirits, but does not set any limits on the content of this compound in distillates of agricultural origin (i.e., raw spirits). Methanol concentrations in the tested distillates (raw spirits) was higher in samples obtained from mashes with pressure-thermal treatment of cereal grains than with the PLS method. The supplementation of mashes with hop α-acid preparation did not cause the changes in the concentration of methyl alcohol in the distillates (Table 7).

#### Principal Component Analysis of Volatile Compounds

The principal component analysis (PCA) analysis of distillates obtained using different methods of starch liberation and saccharification was carried out using concentrations of volatile compounds as variables. To estimate the number of PCA factors, which significantly affect the total variance, a double criterion was used: the own value chart and own values >1. Using the above criteria, the four PCA factors were identified. To isolate PCA1, PCA2, PCA3, and PCA4, the method of normalized varimax rotation was used (Table 8).

The PCA1 accounted for 53.99% of total variance, while the others accounted for 19.56% (PCA2), 12.57% (PCA3), and 9.40% (PCA4). Four principal component factors explained together 95.53% of total variance, which proves that this is a very strong model. In the next step, the value of the charge factors for the four factors was calculated (Table 9). The selection of parameters for the PCA dimension was determined according to charge factors >0.6.

Factors for these dimensions are high. The following assignment to PCA was obtained ((+)—parameter takes higher value; (−)—parameter takes lower value):

− PCA1: (Acetaldehyde (−), Isovaleraldehyde (+), Phenylacetaldehyde (+), 2,3-butanedione (−), Acetaldehyde diethyl acetal (−), Ethyl acetate (+), Ethyl decanoate (+), 2-Phenylethyl isobutyrate (+), 1-propanol (+), 2-methyl-1-propanol (−), 3-methylbutanol (−), 2-methylbutanol (−), 2-phenylethanol).
− PCA2: (Isobutyraldehyde diethyl acetal (+), Ethyl hexanoate (−), 1-butanol).
− PCA3: (Isoamyl acetate (−), Ethyl octanoate (−), Ethyl hexadecanoate (−), Methanol (+)).
− PCA4: (Furfural (+), Isobutyraldehyde (+), Isovaleraldehyde diethyl acetal (+), 2-Methylbutyraldehyde (+), Ethyl 2-hydroxypropanoate (+)).

Due to the character of quantitative PCA parameters and their positive and negative correlations, the Cronbach’s Alpha coefficients were not counted. The data presented in Table 10 summarize the descriptive statistics for all factors of the PCA. Quantitatively, the largest group of volatile compounds determined in the obtained distillates is alcohols. Its concentration was in a broad range. Moreover, in the case of 1-propanol, the standard deviation value was even higher than the mean value. The second abundant group of volatile compounds is esters, where the largest differences in the concentrations were related to ethyl 2-hydroxypropanoate. Similar results have been obtained for some aldehydes (isovaleraldehyde, phenylacetaldehyde, 2-methylbutyraldehyde and furfural), acetals (isovaleraldehyde diethyl acetal and isobutyraldehyde diethyl acetal), and 2,3-butanedione.

In the last part of the analysis, observations were classified based on their correlation to the each PCA factor (Table 11). The PCA4 factor (0.247) is mostly associated to the method of thermal-pressure starch liberation coupled with using of malt enzymes using during starch hydrolysis (thermal pressure method, Munich Malt type II, with addition of α-hop acids). However, due to the fact that it is more closely related to PCA1 (0.557), it has the strongest relationship with this first component.

## 3. Materials and Methods

### 3.1. Materials

The following materials were used in the study:

− barley grain of the Karakan variety (“Danko” Plant Breeding Ltd., Choryń, Poland);
− malted grain of Munich malt type 2 spring barley (Weyermann^®^, Bamberg, Germany);
− dry distillery yeast (*Saccharomyces cerevisiae*) Ethanol Red (Fermentis, a division of S.I. Lesaffre, Marcq en Baroeul Cedex, France) at a dose of 0.5 g d.m./L;
− enzyme preparations: Termamyl S.C. α-amylase preparation was used for liquefaction at a dose of 0.13 mL per 1 kg starch and SAN Extra glucoamylase preparation was used for saccharification at 0.6 mL per 1 kg starch (Novozymes, Bagsværd, Denmark);
− mineral nutrient for yeast—an aqueous solution of (NH_4_)_2_HPO_4_ at a dose of 0.2 g/L mash;
− IsoStab^®^ hop α-acid preparation (BetaTec GmbH, Nürnberg, Germany) as an antimicrobial agent at a dose of 80 ppm.

### 3.2. Analytical Methods

*Starchy raw materials*—both malted and unmalted barley grains were analyzed for starch, reducing sugars, total nitrogen, and moisture content. Starch content was measured using the Ewers polarimetric method [43]. The concentration of reducing sugars was determined using the DNS reagent [44]. Grain humidity was measured in a WPS-305 Radwag weighing dryer (105 °C). Total nitrogen was determined by the Kjeldahl method, calculated as protein (N × 5.7) and expressed as percentage of dry weight [45].

*Sweet and fermented mashes*—the concentration of sugars, ethanol, and soluble solids (expressed as total extract), as well as pH, was determined in mashes before (0 h) and after fermentation (72 h).

Sugar (glucose, maltose, and maltotriose) and ethanol content was determined by HPLC using an Infinity 1260 liquid chromatograph (Agilent Technologies, Santa Clara, CA, USA) with a refractometer detector (RID). The compounds were separated on a Hi-Plex H column (7.7 × 300 mm, 8 μm, Agilent Technologies, USA) at 60 °C using sulfuric acid (H_2_SO_4_, 0.005 M) as a mobile phase with a flow rate of 0.7 mL/min and at an injection volume of 20 µL. The concentration of each compound was determined by measuring the area of the peak in relation to the peak area of the standard solutions (using the external standard method).

The concentration of total sugars (reducing sugars and dextrins after acid hydrolysis) was determined using the DNS reagent and expressed in g glucose/L mash [44]. To determine the degree of starch hydrolysis, dextrin content was calculated as the difference between total sugars and reducing sugars, using a conversion coefficient of 0.9, finally expressed in g/L mash.

The concentration of soluble solids (mostly sugars) in sweet mashes was measured with a hydrometer (results were expressed in g/kg) [24]. Upon completion of the fermentation process, the concentration of soluble solids was determined in mashes after ethanol distillation in a Super Dee digital distilling unit (Gibertini, Novate Milanese, Italy).

*Microbial analysis of mashes*—sweet mashes (0 h), mashes during fermentation (after 24 and 48 h), as well as fermented mashes (after 72 h) were analyzed for yeast [46] (DRBC medium, BTL Ltd., Lodz, Poland; growth conditions: 25 °C, 5 days), LAB (MRS medium, BTL Ltd., Poland; anaerobic growth conditions: 30 °C, 72 h), and total mesophilic bacteria (TMB) [47] (PCA medium, with nystatin, BTL Ltd., Poland; growth conditions: 30 °C, 72 h). Samples of mashes were prepared for microbial analysis according to ISO 6887 [48]. The limit of detection of the above enumeration techniques was 10 cfu/mL. The results were expressed as log cfu/mL.

*Analysis of distillates*—distillates were quantitatively analyzed for volatile compounds by means of gas chromatography using a GC apparatus (Agilent 7890A, Agilent Technologies, Santa Clara, CA, USA) coupled with a mass spectrometer (Agilent MSD 5975C, Agilent Technologies, Santa Clara, CA, USA). A capillary column was used to separate compounds (Agilent VF-WAX MS; 60 m × 0.50 µm × 0.32 mm). The GC oven temperature was programmed from 40 (6 min) to 80 °C at a rate of 2 °C/min, and then increased to 220 °C at a rate of 10 °C/min and maintained for 5 min. The flow rate of the carrier gas (helium) through the column was 2.0 mL/min. The temperature of the injector (split/splitless) was 250 °C. Direct injections of the tested distillates (1 µL) were made in the split mode (1:40). The temperature of the MS ion source, transfer line, and quadrupole was 230 °C, 250 °C, and 150 °C, respectively. The ionization energy was 70 eV.

Identification of the volatile components was based on the comparison of their mass spectra with the mass spectra in the NIST/EPA/NIH Mass Spectra Library (2012; Version 2.0g.). Moreover, retention indices (RIs) were compared with reference compounds and literature data [49,50]. RIs were calculated according to the formula proposed by van den Dool and Kratz [51] relative to a homologous series of n-alkanes from pentane to octadecane. Quantification of the volatile compounds was done using calibration curves in the selected ion monitoring mode (SIM). Six calibration solutions containing different concentrations of each standard compound were prepared with 4-heptanone, which was added at a concentration of 45 mg/L of absolute alcohol to the analyzed samples as an internal standard to monitor instrument response and retention time stability. Quantitative analysis was performed using Agilent MassHunter software (Agilent Technologies, Santa Clara, CA, USA). The results were expressed in mg/L of absolute alcohol.

### 3.3. Preparation of Sweet Mashes

Sweet mashes were prepared both by pressureless starch liberation (PLS) and by the pressure-thermal method.

*PLS method*—mashes were prepared in a cylindrical steel vessel with a depth of 300 mm and an internal diameter of 330 mm (working volume—19 L), equipped with a heating/cooling coil and a thermometer, pursuant to the following procedures:

− mashing with malt enzymes—0.6 kg of barley grain and 0.6 kg of Munich malt type 2 grain was ground and mixed (1:1) with water (3.5 L per 1 kg). The mixture was continuously stirred by an overhead stirrer (CAT, R50) and heated to 53–56 °C. The mash was kept at this temperature for 60 min to conduct starch liquefaction and saccharification (pH was kept at 5.3), and then cooled down to 30 °C.
− mashing with enzyme preparations—1.2 kg of barley grain was ground and mixed with water (3.5 L per 1 kg) previously heated to 50 °C. The mixture was continuously stirred by an overhead stirrer and heated to 90 °C, and then treated with the liquefying Termamyl S.C. preparation. The mixture was kept for 60 min at this temperature (pH was kept at 5.5), then cooled to 65 °C and treated with the saccharifying SAN Extra preparation. Directly after the addition of SAN Extra, the mash was cooled down to 30 °C.

*Pressure-thermal method*—5 kg of barley grain was placed in a tapered cylindrical steamer (cylindrical part dimensions: 210 mm depth and 304 mm internal diameter; tapered part dimensions: 640 mm depth with inclination angle of walls 12°; total volume—40 L; working volume—30 L) previously filled with 17.5 L of water heated to the boiling point, and the steamer was then closed. The raw material was steamed at 150 °C and a pressure of 0.4 MPa for 35 min, with periodical circulation of the content. Upon completion of this step, the content of the steamer was transferred to a cylindrical steel-mashing vessel with a depth of 340 mm and an internal diameter of 300 mm (working volume—19 L), equipped with a heating/cooling coil and a thermometer, and the mashing process was carried out pursuant to the following procedures:

− The steamed mass was continuously stirred by an overhead stirrer and cooled down to 53–56 °C. At the same time, barley Munich malt type 2 was ground and mixed with warm water (heated to 53–56 °C), and the obtained mixture was added to the mashing vessel in a ratio of 1:1 (1 part unmalted grain to 1 part malted grain, *w*/*w*). The mixture was kept at 53–56 °C for 60 min to conduct starch liquefaction and saccharification (pH was kept at 5.3), and then cooled to 30 °C.
− The steamed mass was continuously stirred by an overhead stirrer and cooled to 90 °C, then treated with the liquefying Termamyl S.C. preparation. The mixture was kept for 60 min at this temperature (pH was kept at 5.5), then cooled down to 65 °C and treated with the saccharifying SAN Extra preparation. Immediately after the addition of SAN Extra, the mash was cooled down to 30 °C.

### 3.4. Fermentation Process

Fermentation was carried out using dry distillery yeast Ethanol Red (*Saccharomyces cerevisiae*). Prior to fermentation (i.e., before mashes inoculation), a yeast slurry was prepared according to the procedure described earlier [24] to eliminate undesirable bacterial cells.

The yeast slurry was added to the sweet mash in the amount of 0.5 g of dry yeast per 1 L of mash. The inoculated mashes were also supplemented with diammonium phosphate (0.2 g/L) and finally mixed. The IsoStab^®^ hop α-acid preparation (BetaTec GmbH, Nürnberg, Germany) was added as an inhibitor of microbial infections in selected trials in the amount of 80 mg/L. All fermentation trials were conducted for 3 days, at 35–38 °C; initial pH of mashes was 4.8.

### 3.5. Distillation

Upon completion of the fermentation, ethyl alcohol was distilled from the mashes [24].

### 3.6. Calculations

Fermentation efficiency and total sugar intake were calculated to evaluate the fermentation process [24].

### 3.7. Statistical Analysis

All experiments were performed in triplicate. Statistical analysis was performed using STATISTICA 10.0 software (StatSoft, Tulsa, OK, USA). The obtained results were evaluated using one-way or two-way analysis of variance (ANOVA, at the 0.05 significance level) to indicate differences. If statistical differences were detected (*p* < 0.05), means were compared by Tukey’s test (at the 0.05 significance level).

PCA was used to determine the best differentiation of volatile compounds in the distillates obtained using different methods of starch liberation and saccharification. Moreover, for evaluation of the results of microbial analysis, the discriminant function analysis was used.

## 4. Conclusions

Spirit beverages, such as vodka, whisky, korn, and others, produced from fermented grain mashes are known and appreciated around the world for their organoleptic characteristics. The rising requirements of foods and alcoholic beverages, especially organic products, are the main factors indicating the need to apply technological innovations with a simultaneous monitoring of industrial processes. In the distilling industry, this implies an evaluation of the efficiency of the alcoholic fermentation process and the quality (i.e., appropriate chemical composition and desirable taste and aroma) of the final product (agricultural distillate).

The use of the PLS method in conjunction with malt as a source of amylolytic enzymes is an interesting option, especially in the context of the production of organic spirits. The idea of organic spirits (including vodka) involves a reduction in emissions of harmful pollutants to the environment thanks to novel technological solutions and the use of organic raw materials (cereals, yeast, and enzymes). Nevertheless, special attention should be paid to microbiological purity during production.

The results of our research has shown that the use of malt in the mashing of cereal raw materials, pretreated both by the pressure-thermal and pressureless methods, causes significant contamination of the resulting sweet mashes with lactic acid bacteria. Without the use of antimicrobial protection, bacteria counts are likely to increase, reducing ethanol yield as a result of metabolite (lactic and acetic acids) production and competition for nutrients.

Plant-derived compounds known for their antimicrobial properties against Gram-positive bacteria, such as hop α-acids, can alleviate the presence of LAB, which are the most widespread distillery contaminants. In our study, the antibacterial properties of hop α-acids were shown to reduce LAB content in mashes obtained by the PLS method with malt to less than 4 log cfu/mL and increase ethanol yield by 17% to 78.88 ± 2.69% of the theoretical value, in comparison with the control sample.

The obtained results indicate differences in the concentrations of the volatile compounds in cereal distillates that were mainly affected by a source of amylolytic enzymes applied for starch saccharification during the production of distillery mashes. Distillery mashes prepared from barley mixed with malt resulted in distillates richer in aroma compounds such as esters of fatty acids (ethyl octanoate, ethyl decanoate, and ethyl hexadecanoate) and aldehydes (phenylacetaldehyde). Additionally, bacterial microflora resulted in the formation of aroma-active compound such as ethyl 2-hydroxypropanoate.

## Figures and Tables

**Table 1:** The chemical composition of raw materials.

Components	Content	
	Barley Variety Karakan	Barley Munich Malt Type 2
Moisture (g/kg)	113.20 ± 3.61b	43.40 ± 1.42a
Reducing sugars (g/kg)	71.70 ± 2.40a	184.30 ± 8.43b
Total sugars (g/kg)	682.71 ± 28.42a	736.07 ± 25.51a
Starch (g/kg)	549.90 ± 17.71a	496.52 ± 15.20a
Protein (g/kg d.w.)	97.04 ± 5.13a	95.60 ± 3.33a

Different lower case letters in rows designate statistically significant differences (*p* < 0.05) between means (ANOVA, at a significance level of 0.05). d.w.—dry weight

**Table 2:** Chemical composition of sweet mashes.

Method of Starch Liberation	Source of Amylolytic Enzymes	Extract (g/kg)	pH	Sugars (g/L)				Total Sugars (Glucose) (g/L)	Acids (g/L)	
				Glucose	Maltose	Maltotriose	Dextrins		Lactic Acid	Acetic Acid
PLS	Barley Munich malt type 2	190.64 ± 9.51b	4.8 ± 0.1a	4.06 ± 0.14a	31.26 ± 1.17c	4.88 ± 0.18a	91.12 ± 2.83b	164.70 ± 6.29b	0.04 ± 0.00a	0.08 ± 0.00b
	Termamyl S.C. SanExtra	172.18 ± 7.25a	4.8 ± 0.1a	7.64 ± 0.26b	16.25 ± 0.61b	20.44 ± 0.72c	102.84 ± 3.19c	152.20 ± 3.97a	ND	0.04 ± 0.00a
Pressure-thermal	Barley Munich malt type 2	191.19 ± 11.05b	4.8 ± 0.1a	10.88 ± 0.37c	41.23 ± 1.54d	7.96 ± 0.29b	66.13 ± 2.05a	162.70 ± 4.90b	0.05 ± 0.00a	0.12 ± 0.00b
	Termamyl S.C. SanExtra	175.89 ± 10.08a	4.8 ± 0.1a	35.14 ± 1.20d	9.68 ± 0.31a	21.22 ± 0.76c	87.18 ± 2.82b	150.40 ± 3.69a	ND	0.16 ± 0.00c

Different lower case letters in columns designate statistically significant differences (*p* < 0.05) between fermentation trials (Tukey’s test, at a significance level of 0.05). ND—not detected.

**Table 3:** Chemical composition of fermented mashes.

Method of Starch Liberation	Source of Amylolytic Enzymes/Addition of Hop α-Acids (+ or − *)	Extract (g/kg)	pH	Sugars (g/L)				Total Sugars (Glucose) (g/L)	Acids (g/L)		Ethyl Alcohol (g/L)
				Glucose	Maltose	Maltotriose	Dextrins		Lactic Acid	Acetic Acid	
PLS	Barley Munich malt type 2/−	42.10 ± 1.25f	3.4 ± 0.0a	0.62 ± 0.02f	3.20 ± 0.12c	12.17 ± 0.44e	5.49 ± 0.17b	28.80 ± 0.49ef	7.10 ± 0.21d	0.62 ± 0.01e	53.30 ± 1.82a
	Barley Munich malt type 2/+	29.41 ± 1.09d	4.1 ± 0.1d	0.42 ± 0.01e	3.04 ± 0.11c	4.80 ± 0.18c	2.97 ± 0.09a	9.50 ± 0.36b	0.26 ± 0.01ab	0.22 ± 0.01bc	66.40 ± 2.43d
	Termamyl S.C. SanExtra/−	34.64 ± 1.12e	3.5 ± 0.1a	0.04 ± 0.00a	0.38 ± 0.01b	1.00 ± 0.04b	9.48 ± 0.29d	21.30 ± 0.31cd	0.43 ± 0.01b	0.24 ± 0.01c	59.18 ± 2.19bc
	Termamyl S.C. SanExtra/+	31.05 ± 0.97d	3.6 ± 0.1a	0.04 ± 0.00a	0.40 ± 0.01b	0.96 ± 0.03b	9.99 ± 0.31d	12.10 ± 0.46de	0.19 ± 0.01ab	0.18 ± 0.00b	63.28 ± 2.36c
Pressure-thermal	Barley Munich malt type 2/−	22.34 ± 0.86c	3.3 ± 0.1a	0.08 ± 0.00b	0.18 ± 0.01a	6.34 ± 0.23d	11.95 ± 0.37e	17.30 ± 0.52e	9.58 ± 0.25e	1.72 ± 0.05f	57.72 ± 1.63ab
	Barley Munich malt type 2/+	14.16 ± 0.54b	3.9 ± 0.1c	0.09 ± 0.00b	0.16 ± 0.01a	5.10 ± 0.18c	6.49 ± 0.20c	7.60 ± 0.25a	1.56 ± 0.05c	0.34 ± 0.01d	66.60 ± 2.94d
	Termamyl S.C. SanExtra/−	23.52 ± 1.04c	3.7 ± 0.1bc	0.24 ± 0.01d	0.16 ± 0.01a	0.28 ± 0.01a	14.54 ± 0.45e	16.70 ± 0.41c	0.16 ± 0.00ab	0.19 ± 0.01bc	59.26 ± 2.53bc
	Termamyl S.C. SanExtra/+	12.37 ± 0.75a	3.8 ± 0.1bc	0.19 ± 0.01c	0.12 ± 0.00a	0.22 ± 0.01a	5.83 ± 0.18bc	6.90 ± 0.22a	0.04 ± 0.00a	0.12 ± 0.00a	66.71 ± 2.27d

Different lower case letters in columns designate statistically significant differences (*p* < 0.05) between fermentation trials (Tukey’s test, at a significance level of 0.05). * + (with addition of hop α-acids); − (without addition of hop α-acids).

**Table 4:** Microbiological analysis of mashes.

Method of Starch Liberation	Source of Amylolytic Enzymes/Addition of Hop α-Acids (+ or − *)	Time of Fermentation (h)	Yeast (Y) Count log (cfu/mL)	Lactic Acid Bacteria (LAB) Count log (cfu/mL)	Total Mesophilic Bacteria (TMB) Count log (cfu/mL)
PLS	Barley Munich malt type 2/−	0	6.85 ± 0.44Ca	2.00 ± 0.10Ab	2.72 ± 0.15Ac
		24	8.41 ± 0.40Da	8.12 ± 0.46Be	7.47 ± 0.32Bd
		48	4.18 ± 0.35Ba	7.82 ± 0.62Bf	7.32 ± 0.58Bc
		72	2.54 ± 0.25Aa	7.08 ± 0.63Bc	7.11 ± 0.63Bc
	Barley Munich malt type 2/+	0	6.85 ± 0.44Aa	2.00 ± 0.10Ab	2.72 ± 0.15Ac
		24	8.32 ± 0.41Ba	3.08 ± 0.15Bc	2.61 ± 0.22Aa
		48	8.08 ± 0.34Bb	3.61 ± 0.30Be	3.85 ± 0.30Bb
		72	8.40 ± 0.25Bd	3.34 ± 0.33Bc	3.54 ± 0.31Bb
	Termamyl S.C.; SanExtra/−	0	6.85 ± 0.44Aa	1.00 ± 0.04Aa	1.18 ± 0.06Ab
		24	8.04 ± 0.90Aa	3.85 ± 0.22Bd	4.11 ± 0.22Bb
		48	8.62 ± 0.36Bb	3.30 ± 0.27Be	4.26 ± 0.34Bbc
		72	8.98 ± 0.82Bd	3.04 ± 0.30Bc	3.58 ± 0.31Bb
	Termamyl S.C.; SanExtra/+	0	6.85 ± 0.44Aa	1.00 ± 0.04Aa	1.18 ± 0.06Ab
		24	8.18 ± 0.27Ba	1.78 ± 0.11Bb	2.30 ± 0.13Ca
		48	7.95 ± 0.25Bb	<1.00Aa	1.48 ± 0.11Ba
		72	8.28 ± 0.34Bd	<1.00Aa	1.30 ± 0.11ABa
Pressure-thermal	Barley Munich malt type 2/−	0	6.85 ± 0.44Aa	2.00 ± 0.12Ab	2.46 ± 0.12Ac
		24	8.53 ± 0.70Ba	4.34 ± 0.26Bd	5.11 ± 0.26Bc
		48	7.46 ± 0.52ABb	6.63 ± 0.55Cf	6.71 ± 0.53Cd
		72	6.15 ± 0.47Abc	7.18 ± 0.71Cd	7.87 ± 0.69Cc
	Barley Munich malt type 2/+	0	6.85 ± 0.44Aa	2.00 ± 0.12Cb	2.46 ± 0.12Cc
		24	8.56 ± 0.47Ba	1.12 ± 0.05Ba	2.15 ± 0.12BCa
		48	8.62 ± 0.40Bb	1.48 ± 0.12Bbc	1.90 ± 0.15ABa
		72	8.48 ± 0.42Bd	<1.00Aa	1.70 ± 0.15ABa
	Termamyl S.C.; SanExtra/−	0	6.85 ± 0.40Aa	<1.00Aa	<1.00Aa
		24	7.20 ± 0.27Aa	1.00 ± 0.05Aa	4.40 ± 0.24Cb
		48	8.18 ± 0.27Bb	2.40 ± 0.20Bd	3.32 ± 0.21Bb
		72	7.95 ± 0.37Bcd	3.18 ± 0.31Cc	3.30 ± 0.29Bb
	Termamyl S.C.; SanExtra/+	0	6.85 ± 0.49Aa	<1.00Aa	<1.00Aa
		24	8.43 ± 0.20Ba	<1.00Aa	2.11 ± 0.13Ca
		48	8.34 ± 0.19Bb	1.30 ± 0.11Bb	1.85 ± 0.15BCa
		72	8.04 ± 0.22Bcd	1.18 ± 0.12Bb	1.60 ± 0.13Ba

Different capital letters in columns for each fermentation variant designate statistically significant differences (*p* < 0.05) between means (Tukey’s test, at a significance level of 0.05). Different lower case letters in columns designate statistically significant differences (*p* < 0.05) between fermentation variants at the same time (Tukey’s test, at a significance level of 0.05). * + (with addition of hop α-acids); − (without addition of hop α-acids).

**Table 5:** Ethanol fermentation factors.

Method of Starch Liberation	Source of Amylolytic Enzymes/Addition of Hop α-Acids (+ or − *)	Intake of Total Sugars (%)	Fermentation Efficiency (% of Theoretical)
PLS	Barley Munich malt type 2/−	92.23 ± 2.77a	63.32 ± 2.16a
	Barley Munich malt type 2/+	94.23 ± 2.84a	78.88 ± 2.69bc
	Termamyl S.C.; SanExtra/−	92.58 ± 3.21a	76.08 ± 2.59bc
	Termamyl S.C.; SanExtra/+	92.05 ± 3.78a	81.35 ± 2.77d
Pressure-thermal	Barley Munich malt type 2/−	91.58 ± 3.29a	69.41 ± 2.37a
	Barley Munich malt type 2/+	95.33 ± 4.21a	80.09 ± 3.53cd
	Termamyl S.C.; SanExtra/−	93.07 ± 3.45a	75.09 ± 3.20bc
	Termamyl S.C.; SanExtra/+	95.53 ± 3.21a	84.53 ± 2.88d

Different lower case letters in columns designate statistically significant differences (*p* < 0.05) between fermentation variants (Tukey’s test, at a significance level of 0.05). * + (with addition of hop α-acids); − (without addition of hop α-acids).

**Table 6:** Results of discriminant function analysis.

Variable	Microorganisms log (cfu/mL)	Significance of the Model and Discrimination	Wilks’ Lambda *	Wilks’ Part. *	F = 1.92 *	*p* *
Method of starch liberation	Y	Wilks’ Lambda: 0.89085 F(3.92) = 3.7573 *p* < 0.0135	0.900	0.990	0.902	0.345
	LAB		**0.979**	**0.910**	**9.134**	**0.003**
	TMB		**0.950**	**0.937**	**6.138**	**0.015**
Source of amylolytic enzyme	Y	Wilks’ Lambda: 0.01323 F(45.232) = 16.992 *p* < 0.0135	**0.036**	**0.365**	**9.035**	**<0.01**
	LAB		**0.026**	**0.505**	**5.100**	**<0.01**
	TMB		**0.056**	**0.238**	**16.656**	**<0.01**
Time of fermentation	Y	Wilks’ Lambda: 0.525 F (9.219) = 7.3776 *p* < 0.01	**0.807**	**0.650**	**16.121**	**<0.01**
	LAB		0.532	0.988	0.374	0.772
	TMB		**0.597**	**0.880**	**4.108**	**0.009**
Method of starch liberation × Source of amylolytic enzymes	Y	Wilks’ Lambda: 0.21241 F(21.247) = 8.4290 *p* < 0.01	**0.292**	**0.726**	**4.629**	**<0.01**
	LAB		**0.341**	**0.623**	**7.436**	**<0.01**
	TMB		**0.274**	**0.775**	**3.573**	**0.002**
Method of starch liberation × Time of fermentation	Y	Wilks’ Lambda: 0.31851 F(21.247) = 5.7690 *p* < 0.01	**0.504**	**0.631**	**7.170**	**<0.01**
	LAB		**0.503**	**0.633**	**7.118**	**<0.01**
	TMB		**0.511**	**0.623**	**7.425**	**<0.01**
Source of amylolytic enzymes × Time of fermentation	Y	Wilks’ Lambda: 0.01323 F(45.232) = 16.992 *p* < 0.01	**0.036**	**0.365**	**9.035**	**<0.01**
	LAB		**0.026**	**0.505**	**5.100**	**<0.01**
	TMB		**0.056**	**0.238**	**16.656**	**<0.01**
Method of starch liberation × Source of amylolytic enzymes × Time of fermentation	Y	Wilks’ Lambda: 0.00001 F(93.186) = 114.75 *p* < 0.01	**0.000**	**0.074**	**24.856**	**<0.01**
	LAB		**0.001**	**0.004**	**554.449**	**<0.01**
	TMB		**0.001**	**0.004**	**505.327**	**<0.01**

Y—yeast; LAB—lactic acid bacteria; TMB—total mesophilic bacteria. * The values of the discriminant function in bold are statistically significant.

**Table 7:** Chemical composition of the obtained distillates.

Volatile Compounds	Method of Starch Liberation & Source of Enzymes							
	PLS				Pressure-thermal			
	Enzymes: Munich Malt Type 2 without Addition of Hop α-Acids	Enzymes: Munich Malt Type 2 with Addition of Hop α-Acids	Enzymes: Termamyl S.C.; SanExtra without Addition of Hop α-Acids	Enzymes: Termamyl S.C.; SanExtra with Addition of Hop α-Acids	Enzymes: Munich Malt Type 2 without Addition of Hop α-Acids	Enzymes: Munich Malt Type 2 with Addition of Hop α-Acids	Enzymes: Termamyl S.C.; SanExtra without Addition of Hop α-Acids	Enzymes: Termamyl S.C.; SanExtra with Addition of Hop α-Acids
CARBONYL COMPOUNDS	(mg/L of absolute alcohol)							
Acetaldehyde	34.247 ± 0.982e	12.842 ± 0.357b	41.952 ± 1.187f	34.247 ± 0.997e	17.123 ± 0.511c	10.098 ± 0.239a	29.966 ± 0.758d	15.103 ± 0.444bc
Furfural	225.807 ± 6.475e	79.700 ± 2.213b	43.532 ± 1.231a	49.774 ± 1.448a	271.249 ± 8.090f	120.366 ± 2.851d	95.501 ± 2.416c	50.400 ± 1.482a
Isobutyraldehyde	4.214 ± 0.121d	2.536 ± 0.070b	3.567 ± 0.101c	1.866 ± 0.054a	6.233 ± 0.186f	2.107 ± 0.050a	5.611 ± 0.142e	1.754 ± 0.052a
Isovaleraldehyde	14.632 ± 0.420c	10.302 ± 0.286b	ND	ND	61.866 ± 1.845e	40.214 ± 0.952d	ND	ND
2-Methylbutyraldehyde	5.348 ± 0.153c	3.268 ± 0.091b	8.211 ± 0.232d	5.765 ± 0.168c	21.898 ± 0.653f	9.412 ± 0.223e	3.416 ± 0.086b	1.997 ± 0.059a
Phenylacetaldehyde	6.766 ± 0.194	3.819 ± 0.106b	ND	ND	11.322 ± 0.338e	7.895 ± 0.187d	ND	ND
2,3-Butanedione	8.416 ± 0.241c	6.188 ± 0.172ab	53.537 ± 1.514e	40.783 ± 1.187d	6.722 ± 0.200ab	3.455 ± 0.082a	64.029 ± 1.620f	30.841 ± 0.907c
ACETALS	(mg/L of absolute alcohol)							
Acetaldehyde diethyl acetal	70.334 ± 2.017a	63.823 ± 1.772a	214.836 ± 6.077e	176.780 ± 5.145d	112.472 ± 3.355b	101.720 ± 2.409b	154.424 ± 3.907c	109.086 ± 3.207b
Isobutyraldehyde diethyl acetal	1.872 ± 0.054e	0.917 ± 0.025b	3.487 ± 0.099d	3.494 ± 0.102d	6.153 ± 0.184f	1.296 ± 0.031c	ND	ND
Isovaleraldehyde diethyl acetal	4.276 ± 0.123b	4.233 ± 0.118b	ND	ND	9.915 ± 0.296c	ND	ND	ND
ESTERS	(mg/L of absolute alcohol)							
Ethyl acetate	502.371 ± 14.405c	498.964 ± 13.854c	282.430 ± 7.989a	306.258 ± 8.913a	953.045 ± 28.425d	962.153 ± 22.787d	417.976 ± 10.576b	410.906 ± 12.080b
Isoamyl acetate	3.448 ± 0.099e	3.792 ± 0.105d	3.618 ± 0.102c	3.822 ± 0.111c	2.983 ± 0.089c	2.912 ± 0.069b	2.811 ± 0.071b	2.735 ± 0.080a
Ethyl hexanoate	2.916 ± 0.084b	3.038 ± 0.084b	2.234 ± 0.063a	1.977 ± 0.058a	2.035 ± 0.061a	1.989 ± 0.047a	6.205 ± 0.157d	5.289 ± 0.155c
Ethyl octanoate	11.512 ± 0.330d	10.616 ± 0.295b	8.393 ± 0.237c	7.627 ± 0.222b	7.907 ± 0.236a	6.746 ± 0.160a	6.349 ± 0.161b	5.103 ± 0.150b
Ethyl decanoate	1.708 ± 0.049b	1.725 ± 0.048b	1.399 ± 0.040a	1.356 ± 0.039a	2.022 ± 0.060c	1.921 ± 0.045c	1.334 ± 0.034a	1.256 ± 0.037a
Ethyl hexadecanoate	8.642 ± 0.248d	3.669 ± 0.102b	6.202 ± 0.175c	3.909 ± 0.114b	1.179 ± 0.035a	1.202 ± 0.028a	3.535 ± 0.089b	3.518 ± 0.103b
2-Phenylethyl isobutyrate	2.178 ± 0.062d	2.055 ± 0.057d	1.527 ± 0.043b	1.481 ± 0.043b	2.044 ± 0.061d	1.879 ± 0.045c	1.153 ± 0.029a	1.088 ± 0.032a
Ethyl 2-hydroxypropanoate	96.540 ± 2.768c	ND	ND	ND	140.889 ± 4.202d	ND	4.028 ± 0.102b	ND
ALCOHOLS	(mg/L of absolute alcohol)							
1-Propanol	1065.428 ± 30.551b	1416.376 ± 39.326c	674.938 ± 19.093a	589.248 ± 17.148a	10,536.890 ± 314.267d	10,521.101 ± 249.172d	1648.119 ± 41.702c	1625.719 ± 47.792c
2-Methyl-1-propanol	5287.157 ± 151.606b	5626.924 ± 156.231b	10,590.344 ± 299.582d	10,111.506 ± 294.259d	3220.649 ± 96.057a	3204.551 ± 75.894a	7071.816 ± 178.935c	7013.669 ± 206.185c
1-Butanol	34.869 ± 1.000c	39.943 ± 1.109d	15.869 ± 0.449b	12.614 ± 0.367a	30.819 ± 0.919b	30.623 ± 0.725b	36.664 ± 0.928c	36.702 ± 1.079c
3-Methylbutanol	6936.398 ± 198.897b	7324.607 ± 203.367b	12,883.438 ± 364.449d	13,562.804 ± 394.696d	4980.179 ± 148.536a	4892.402 ± 115.867a	11,712.660 ± 296.361c	11,710.791 ± 344.270c
2-Methylbutanol	3398.718 ± 97.456b	3634.696 ± 100.917b	5058.071 ± 143.084c	5199.437 ± 151.311c	1926.010 ± 57.444a	1900.361 ± 45.006a	4969.325 ± 125.737c	4971.144 ± 146.140c
2-Phenylethanol	803.119 ± 23.029b	941.135 ± 26.131b	2603.190 ± 73.640d	2629.700 ± 76.528d	266.199 ± 7.939a	262.343 ± 6.213a	1150.156 ± 29.102c	1117.587 ± 32.854c
Methanol	57.906 ± 1.660b	55.679 ± 1.546b	35.635 ± 1.008a	33.408 ± 0.972a	80.178 ± 2.391d	80.185 ± 1.899d	73.497 ± 1.860c	73.483 ± 2.160c

Different lower case letters in rows designate statistically significant differences (*p* < 0.05) between means (Tukey’s test, at a significance level of 0.05). ND—not detected.

**Table 8:** Factor loadings of main four principal components for normalized varimax rotation.

PCA Factor	Own Value	% of Variance	The Cumulated Own Value	Cumulative %
PCA1	13.50	53.99	13.50	53.99
PCA2	4.89	19.56	18.39	73.56
PCA3	3.14	12.57	21.53	86.13
PCA4	2.35	9.40	23.88	95.53

**Table 9:** Values of the charge factors >0.6.

Compound	PCA1	PCA2	PCA3	PCA4
Acetaldehyde	**−0.701**	0.319	−0.501	0.248
Furfural	0.545	−0.068	−0.034	**0.810**
Isobutyraldehyde	−0.083	−0.150	0.063	**0.931**
Isovaleraldehyde	**0.721**	0.210	0.415	0.513
2-Methylbutyraldehyde	0.370	0.486	0.371	**0.688**
Phenylacetaldehyde	**0.833**	0.096	0.134	0.519
2,3-Butanedione	**−0.971**	−0.001	0.085	−0.035
Acetaldehyde diethyl acetal	**−0.793**	0.544	0.182	0.009
Isobutyraldehyde diethyl acetal	0.131	**0.761**	0.035	0.596
Isovaleraldehyde diethyl acetal	0.547	0.057	−0.105	**0.717**
Ethyl acetate	**0.780**	0.041	0.519	0.317
Isoamyl acetate	−0.002	0.547	**−0.755**	−0.230
Ethyl hexanoate	−0.493	**−0.843**	0.176	−0.041
Ethyl octanoate	0.400	0.101	**−0.869**	0.202
Ethyl decanoate	**0.887**	0.178	0.101	0.377
Ethyl hexadecanoate	−0.305	−0.099	**−0.856**	0.081
2-Phenylethyl isobutyrate	**0.818**	0.225	−0.419	0.299
Ethyl 2-hydroxypropionate	0.386	0.042	−0.098	**0.874**
1-Propanol	**0.643**	0.183	**0.664**	0.306
2-Methyl−1-propanol	**−0.834**	0.343	−0.301	−0.300
1-Butanol	0.420	**−0.881**	0.033	0.079
3-Methylbutanol	**−0.927**	0.113	−0.107	−0.307
2-Methylbutanol	**−0.891**	−0.071	−0.254	−0.331
2-Phenylethanol	**−0.744**	0.507	−0.316	−0.292
Methanol	0.439	−0.575	**0.630**	0.272

The PCA1, PCA2, PCA3, and PCA4 variables are marked in bold.

**Table 10:** Descriptive statistics for determined volatile compounds grouped into principal component analysis (PCA) factors.

Dimension	Compound	Mean	Median	Minimum	Maximum	Standard Deviation
PCA1	Acetaldehyde	24.45	23.54	10.10	41.95	12.01
	Isovaleraldehyde	15.88	5.15	0.00	61.87	23.14
	Phenylacetaldehyde	3.73	1.91	0.00	11.32	4.47
	2,3-Butanedione	26.75	19.63	3.46	64.03	23.98
	Acetaldehyde diethyl acetal	125.43	110.78	63.82	214.84	52.51
	Ethyl acetate	541.76	458.47	282.43	962.15	268.44
	Ethyl decanoate	1.59	1.55	1.26	2.02	0.29
	2-Phenylethyl isobutyrate	1.68	1.70	1.09	2.18	0.42
	1-Propanol	3509.73	1521.05	589.25	10,536.89	4350.33
	2-Methyl-1-propanol	6515.83	6320.30	3204.55	10,590.34	2782.86
	3-Methylbutanol	9250.41	9517.70	4892.40	13,562.80	3589.71
	2-Methylbutanol	3882.22	4302.01	1900.36	5199.44	1390.52
	2-Phenylethanol	1221.68	1029.36	262.34	2629.70	925.37
PCA2	Isobutyraldehyde diethyl acetal	2.15	1.58	0.00	6.15	2.11
	Ethyl hexanoate	3.21	2.58	1.98	6.21	1.64
	1-Butanol	29.76	32.84	12.61	39.94	10.10
PCA3	Isoamyl acetate	3.27	3.22	2.74	3.82	0.45
	Ethyl octanoate	8.03	7.77	5.10	11.51	2.14
	Ethyl hexadecanoate	3.98	3.60	1.18	8.64	2.47
	Methanol	61.25	65.69	33.41	80.19	18.86
PCA4	Furfural	117.04	87.60	43.53	271.25	86.06
	Isobutyraldehyde	3.49	3.05	1.75	6.23	1.73
	2-Methylbutyraldehyde	7.41	5.56	2.00	21.90	6.37
	Isovaleraldehyde diethyl acetal	2.30	0.00	0.00	9.92	3.63
	Ethyl 2-hydroxypropionate	30.18	0.00	0.00	140.89	55.93

**Table 11:** Squared cosines of the observations.

Method of Starch Liberation & Saccharification	PCA1	PCA2	PCA3	PCA4
PLS (source of enzymes—malt) Without addition of hop α-acids	0.146	0.176	**0.451**	0.163
PLS (source of enzymes—malt) With addition of hop α-acids	0.047	0.001	**0.695**	0.097
PLS (source of enzymes—enzyme preparations) Without addition of hop α-acids	**0.661**	0.264	0.052	0.000
PLS (source of enzymes—enzyme preparations) With addition of hop α-acids	**0.666**	0.198	0.033	0.080
Thermal-pressure (source of enzymes—malt) Without addition of hop α-acids	**0.836**	0.035	0.096	0.021
Thermal-pressure (source of enzymes—malt) With addition of hop α-acids	**0.557**	0.127	0.012	0.247
Thermal-pressure (source of enzymes—enzyme preparations) Without addition of hop α-acids	0.308	**0.347**	0.039	0.260
Thermal-pressure (source of enzymes—enzyme preparations) With addition of hop α-acids	0.273	**0.641**	0.000	0.000

Values in bold correspond for each observation to the factor for which the squared cosine is the largest.
